# Endothelial cell telomere dysfunction induces senescence and results in vascular and metabolic impairments

**DOI:** 10.1111/acel.13875

**Published:** 2023-05-31

**Authors:** Samuel I. Bloom, Yu Liu, Jordan R. Tucker, Md Torikul Islam, Daniel R. Machin, Hossein Abdeahad, Tyler G. Thomas, R. Colton Bramwell, Lisa A. Lesniewski, Anthony J. Donato

**Affiliations:** ^1^ Department of Nutrition and Integrative Physiology The University of Utah Salt Lake City Utah USA; ^2^ Department of Geriatrics Tongji Hospital Wuhan China; ^3^ Division of Geriatrics, Department of Internal Medicine University of Utah School of Medicine Salt Lake City Utah USA; ^4^ Department of Nutrition and Integrative Physiology Florida State University Tallahassee Florida USA; ^5^ Geriatric Research, Education and Clinical Center Veteran's Affairs Medical Center‐Salt Lake City Salt Lake City Utah USA; ^6^ Nora Eccles Harrison Cardiovascular Research and Training Institute The University of Utah Salt Lake City Utah USA; ^7^ Department of Biochemistry The University of Utah Salt Lake City Utah USA

**Keywords:** aging, endothelial cell, metabolic function, senescence, telomeres, vascular function

## Abstract

In advanced age, increases in oxidative stress and inflammation impair endothelial function, which contributes to the development of cardiovascular disease (CVD). One plausible source of this oxidative stress and inflammation is an increase in the abundance of senescent endothelial cells. Cellular senescence is a cell cycle arrest that occurs in response to various damaging stimuli. In the present study, we tested the hypothesis that advanced age results in endothelial cell telomere dysfunction that induces senescence. In both human and mouse endothelial cells, advanced age resulted in an increased abundance of dysfunctional telomeres, characterized by activation of DNA damage signaling at telomeric DNA. To test whether this results in senescence, we selectively reduced the telomere shelterin protein telomere repeat binding factor 2 (*Trf2*) from endothelial cells of young mice. *Trf2* reduction increased endothelial cell telomere dysfunction and resulted in cellular senescence. Furthermore, induction of endothelial cell telomere dysfunction increased inflammatory signaling and oxidative stress, resulting in impairments in endothelial function. Finally, we demonstrate that endothelial cell telomere dysfunction‐induced senescence impairs glucose tolerance. This likely occurs through increases in inflammatory signaling in the liver and adipose tissue, as well as reductions in microvascular density and vasodilation to metabolic stimuli. Cumulatively, the findings of the present study identify age‐related telomere dysfunction as a mechanism that leads to endothelial cell senescence. Furthermore, these data provide compelling evidence that senescent endothelial cells contribute to age‐related increases in oxidative stress and inflammation that impair arterial and metabolic function.

## Graphical abstract

The present study provides important evidence that aging results in an increased abundance of endothelial cells with dysfunctional telomeres in humans and mice. Moreover, we demonstrate that genetic induction of telomere dysfunction specifically in endothelial cells results in cellular senescence and exacerbates multiple hallmarks of vascular and metabolic aging.

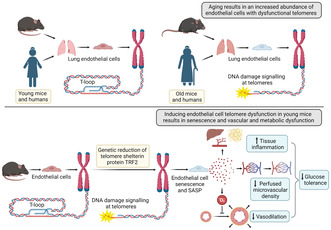

## 1 INTRODUCTION

Advanced age is the greatest risk factor for the development of cardiovascular diseases (CVDs), which are the leading cause of death (National Center for Health, US, 2017). With advancing age, oxidative stress and inflammation are elevated which induces endothelial dysfunction (Donato et al., 2018). Importantly, endothelial dysfunction precedes and predicts future CVD morbidity and mortality (Donato et al., 2018). However, the mechanisms responsible for increased oxidative stress and inflammation that lead to endothelial dysfunction are incompletely understood.

Increases in the abundance of senescent cells within arteries represent a source of oxidative stress and inflammation that contribute to endothelial dysfunction (Bloom, Tucker, et al., 2022; Liu et al., 2019). Cellular senescence is a cell cycle arrest that occurs in response to a variety of damaging stimuli (Childs et al., 2015). By preventing damaged cells from dividing, cellular senescence acts as a potent anticancer mechanism; however, senescent cells adopt a pro‐oxidative pro‐inflammatory phenotype known as the senescence‐associated secretory phenotype (SASP) (Childs et al., 2015). Through autocrine, paracrine, and endocrine effects of the SASP, senescent cells contribute to age‐related physiological dysfunction and disease (Childs et al., 2015). Accordingly, reducing the senescence burden with drugs that selectively kill senescent cells, known as senolytics, has shown great translational promise to treat many age‐related diseases (Chaib et al., 2022; Gasek et al., 2021). Many senolytic drugs have at least some degree of cell type specificity (Zhu et al., 2015; Zhu et al., 2017). Therefore, to develop effective therapeutics, it is important to identify which cell types to target in the context of physiological dysfunction, aging, and disease.

Mounting evidence suggests that endothelial cell senescence contributes to age‐related endothelial dysfunction (Bloom, Islam, et al., 2022; Liu et al., 2019). In older adults, endothelial cell senescence is associated with impaired endothelium‐dependent dilation (EDD) (Rossman et al., 2017), and in old mice, systemic administration of senolytics improves EDD (Roos et al., 2016). Additionally, senescent endothelial cells may contribute to physiological dysfunction not traditionally thought of as vascular in origin. For example, senescent endothelial cells could impair systemic metabolism because endothelial cells reside within every tissue and therefore can act as a local source of inflammation in metabolically active tissues (Hasegawa et al., 2012). Another possible way that endothelial cell senescence could impair metabolic function is by altering the delivery of nutrients via changes in vascular density or dilation to metabolic stimuli (Bloom, Islam, et al., 2022). However, there is a paucity of evidence identifying mechanisms responsible for the induction of endothelial cell senescence in advanced age and the settings in which endothelial cell senescence is deleterious.

One likely inducer of endothelial cell senescence in advancing age is telomere dysfunction (Morgan et al., 2018). Telomeres are repeat sequences at the ends of chromosomes that shorten with each cell division and are highly sensitive to oxidative modifications within their guanine‐rich sequence (TTAGGG) (Barnes et al., 2022; de Lange, 2009; Oikawa & Kawanishi, 1999; Opresko et al., 2005). Excessive shortening and oxidation can impair the formation of the telomere loop (T‐loop), which caps chromosome ends (Barnes et al., 2022; Nassour et al., 2021; Opresko et al., 2005). Additionally, loss of the telomere‐associated shelterin protein known as telomere repeat binding factor 2 (TRF2) can result in uncapping (Doksani et al., 2013; Van Ly et al., 2018). Following uncapping, telomeres are recognized by the DNA damage response (DDR) machinery, resulting in DDR signaling at telomeres known as telomere dysfunction‐induced foci (TIF) (de Lange, 2009; Nassour et al., 2021). TIF are largely irreparable, leading to persistent DDR signaling and cellular senescence (Fumagalli et al., 2012; Hewitt et al., 2012; Morgan et al., 2019). In total, due to their sensitivity to both replicative and oxidative stress, telomere dysfunction can induce premature or replicative senescence, and therefore telomeres act as cellular stress sensors (Victorelli & Passos, 2017). Here, we test the hypothesis that aging results in endothelial cell telomere dysfunction (i.e., formation of TIF) and that induction of telomere dysfunction specifically in endothelial cells phenocopies age‐related arterial and metabolic dysfunction.

## 2 METHODS

### 2.1 Ethical approval and animal studies

All animal studies were in compliance with the Guide and Use of Laboratory Animals and were approved by the University of Utah, Veteran's Affairs Medical Center – Salt Lake City (VAMC‐SLC). Young 2.7 ± 0.0 mo C57BL/6J male mice (*N* = 12) were obtained from Charles River Inc. Old 27.3 ± 0. 2 mo C57Bl/6J mice (*N* = 12) were obtained from the National Institute of Aging colony maintained by Charles River Inc. Mice containing tamoxifen‐inducible Cdh5‐Cre (otherwise known as VE‐Cadherin) were crossed with mice containing LoxP sites flanking Exons 1 and 2 of the *Trf2* gene. Young 3.6 ± 0.2 mo male and female *Trf2* ^f/f^ Cdh5‐Cre + (EC‐TRF2^f/f^, *N* = 23) mice were compared to young littermate 3.5 ± 0.1 mo male and female *Trf2* ^+/+^Cdh5‐Cre+ (WT, *N* = 25) mice. All mice received one injection of 0.05 mg tamoxifen in 50 μL corn oil on postnatal Day 1.

### 2.2 Primary lung endothelial cell isolation and evaluation

Mouse lungs were dissected and placed into cold isolation media containing DMEM, 20% FBS, and 1% penicillin–streptomycin. Lungs were then mechanically digested using sterile scissors and placed into sterilized dissociation media containing DMEM and 2 mg/mL Collagenase Type 2 at 37°C for 40 min. Lungs were then titrated through a 14‐gauge metal cannula until dissociated into a single cell suspension, passed through a 70 μm cell strainer, and washed in isolation media to stop digestion. Samples were spun at 1000 rpm for 10 min at 4°C. Following centrifugation, the supernatant was removed and cells were resuspended in 2 mL sterile 1x PBS containing 0.1% BSA. Cells were then tumbled with anti‐PECAM (CD‐31) beads bound to sheep anti‐rat IgG Dynabeads for 30 min and cells were isolated using a magnetic separator. For IF‐FISH experiments, cells were cultured until 40%–60% confluent and fixed in 4% paraformaldehyde pH 7.4. For assessments of SA‐βgal, BrdU, and cell migration, cells were trypsinized, tumbled with anti‐ICAM2 (CD‐102) beads for 30 min, and isolated with a magnetic separator. Cells were grown until confluent prior to experimentation. For comparisons of telomere dysfunction using immunofluorescence fluorescent in situ hybridization (IF‐FISH) in lung endothelial cells, lungs from three mice were pooled to perform two experiments. For all other experiments, lungs from three mice were pooled for each individual experiment. For experiments using WT (*N* = 9 mice/experiment) and EC‐TRF2^f/f^ mice (*N* = 9 mice/experiment), cells were treated for 24 h in 0.6 μM 4‐hydroxytamoxifen (4‐OHT) followed by 24 h recovery before assessment.

Human lung microvascular endothelial cells were purchased from Lonza from two young donors (29 ± 1 yo) and two old donors (67 ± 1 yo). Sixty‐four cells were analyzed from young humans averaging 16 cells per experiment, and 57 from old humans, averaging 19 cells per experiment. Individual data points represent averages from experimental replicates.

Cells were plated onto fibronectin‐coated glass coverslips and grown until 40%–60% confluent, fixed in 4% paraformaldehyde pH 7.4 for 15 min at room temperature, and then stored in 1x PBS at 4°C overnight prior to IF‐FISH.

For evaluation of SA‐βgal, cells were fixed in 2% paraformaldehyde pH 7.4 containing 0.25% glutaraldehyde in 1x PBS, pH 7.3 for 1 h. Cells were then stained for 18 h at 37°C in a fresh solution containing 40 mM citric acid/sodium phosphate buffer, 5 mM potassium hexacyanoferrate, 5 mM potassium hexacyanoferrate trihydrate, 150 mM sodium chloride, 2 mM magnesium chloride, and 1 mg/mL Xgal solution containing 20 mg of Xgal in 1 mL of *N*,*N*‐dimethylformamide.

For evaluation of BrdU cell proliferation, cells were treated according to the manufacturer's protocols. For wound healing (scratch migration) assays, a 10‐mm scratch was made using a sterile pipette tip and cell migration was tracked over the next 24 h. For evaluation of barrier function, a 96‐well electric cell substrate impedance sensing (ECIS) plate was used. Lung endothelial cells were plated at equal density (4 × 10^4^/well) and plates were monitored using the ECIS Zθ system (Applied Biophysics). Resistance of an alternating current (4000 Hz) across the endothelial cell monolayer was measured. Twenty‐four hours into measurement, cells were treated with 0.6 μM 4‐OHT for 24 h and then replaced by fresh medium. Resistance values were normalized for each well to the value recorded at the time fresh medium was added.

To evaluate telomere dysfunction, IF‐FISH was performed. Briefly, cells were fixed on glass coverslips in 4% paraformaldehyde pH 7.4 for 15 min, then rehydrated in 1x PBS for 5 min all at room temperature. Coverslips were then placed in 100% methanol for 15 min at −20°C, rehydrated in 1x PBS for 5 min, and incubated in a blocking solution containing 1 mg/L BSA, 3% goat serum, 0.1% Triton X 100, 1 mM EDTA, all in 1x PBS. Cells were incubated in 1:500 anti 53BP1 antibody (Novus Biologicals, NB100‐304, Rabbit) in blocking solution for 1 h, washed 3x 5 min in 1x PBS, incubated with 1:500 AF 555 (Invitrogen, A‐21429, Goat Anti‐Rabbit) in blocking solution, and washed 3x 5 min in 1x PBS, all at room temperature. Cells were then dehydrated in 70%, 95%, and 100% ethanol for 5 min each. Coverslips were allowed to dry briefly, and then 50 μL of hybridization solution containing 2% Tris HCL, 60% formamide, 5% blocking reagent from a 10% Roche stock, and 1:200 Tel Probe (Integrated DNA Technologies, 5Alex488N/CC CTA ACC CTA ACC CTA A, purification: HPLC) was added. Coverslips were then incubated at 60°C for 10 min, 85°C for 10 min, and 2 h at 37°C, followed by washing 2x 10 min at 60°C in 1x PBS containing 2x saline‐sodium citrate (SSC) and 0.1% Tween 20. Coverslips were then washed in 2x SSC, 1x SSC, and diH_2_O for 10 min at room temperature, mounted with DAPI Fluoromount G (VWR—Catalog #102092‐102), and weighted with 1 kg weight for 5 min and stored in a dark container.

To determine endothelial mitochondrial superoxide levels, cells were cultured until confluent and then stained with MitoSOX (Invitrogen Catalog #M36008) according to the manufacturer's protocol. Cells were then mounted with DAPI Fluoromount G (VWR – Catalog #102092‐102) and stored in a dark container until imaging.

### 2.3 Imaging and analysis

IF‐FISH samples were imaged on an Olympus Fluoview FV1000 confocal microscope at 100x magnification, and all samples were imaged with the same acquisition settings. One micrometer Z‐slices were taken through each nucleus and Z‐stacked using Fiji is Just ImageJ (FIJI). Images were converted to 16‐bit images and telomere dysfunction was identified as discrete 53BP1 foci that colocalized with telomere signal. Telomere length was ascertained using the Telometer Plugin for ImageJ (https://demarzolab.pathology.jhmi.edu/telometer/index.html) from mean telomere fluorescence intensity, which is calculated by the Telometer as the sum of all fluorescent intensity values for all pixels of the object divided by the object's area.

MitoSOX samples were imaged at 40x magnification and an equal number of 1 μm z‐slices were collected for each sample. Images were analyzed using FIJI is Just ImageJ and mitochondrial superoxide levels was quantified from thresholded images using the integrated density measure.

### 2.4 Perfused microvascular density measurement

Perfused microvascular density was evaluated as described previously (Machin et al., 2018). In brief, mice were anesthetized under 2% isoflurane anesthesia and 98% oxygen in the supine position on a warming platform. With sterile scissors, a midline incision was made and blunt forceps were used to gently pull the intestines from the abdominal cavity into a 37°C isotonic 0.9% saline bath. The cecum was identified and imaged using an intravital microscope and the GlycoCheck Measurement System™ which automatically identifies and records 3000 vascular segments and calculates the number of perfused microvessels between 5 and 25 μm. Five to 10 videos of different areas were taken per mouse, and the average value of all videos was used for analysis.

### 2.5 Ex vivo vascular function assessments

To examine EDD, the mesenteric arcade was excised following perfused microvascular density measurements, placed in cold physiological saline solution, and mesenteric arteries were dissected, cleaned of perivascular adipose, and cannulated on a pressure myograph (DMT Inc.) under a dissecting microscope. Arteries were pressurized to 68 cmH_2_O, preconstricted with phenylephrine, and then EDD was assessed by cumulative addition of acetylcholine (ACh, 10^−9^ to 10^−4^ M) and insulin (0.01–10 nM) in the absence and presence of the nitric oxide synthase inhibitor l‐NAME (0.1 nM, 30 min). To examine superoxide suppression of EDD, responses to ACh in the absence and presence of l‐NAME were repeated in the presence of the superoxide scavenger TEMPOL (1 mM, 1 h). To evaluate endothelium‐independent dilation, responses to the exogenous nitric oxide (NO) donor sodium nitroprusside (SNP) were performed (10^−10^ to 10^−4^ M).

### 2.6 Superoxide levels

To evaluate arterial superoxide levels, carotid arteries were cleared of perivascular tissue and 2 mm sections were isolated. Arterial sections were washed one time in cold PSS and then incubated for 1 h at 37°C in modified Krebs‐HEPES buffer containing 0.5 mmol L^−1^ CMH. Samples were then snap‐frozen in liquid nitrogen and stored at −80°C until analysis. Samples were analyzed on an MS300 X‐band EPR Spectrometer (Magnettech) using the following settings: microwave frequency 9.83 GHz, centerfield 3480 G, sweep 80 G, modulation amplitude 3.3 G, microwave power 40 mW, microwave attenuation 7, and receiver gain 30. A total of six sweeps were conducted lasting 8.7 s per sweep. The running average of the six sweeps was collected with the double integration (area under and over the baseline) of the triplet used to display the magnitude of the signal. The magnitude of this signal directly relates to the amount of superoxide that has been trapped by the CMH. All values were normalized to WT superoxide levels.

### 2.7 qRT‐PCR

Total RNA was isolated from primary lung endothelial cells, carotid artery endothelial cell effluent, mesenteric arteries, perigonadal adipose tissue, quadriceps skeletal muscle, and liver using the RNAeasy Mini Kit (Qiagen) according to the manufacturer's protocol. Mesenteric arteries from two mice from the same group were pooled to obtain sufficient RNA yields. mRNA was converted to cDNA using QuantiTect Reverse Transcription Kit (Qiagen) according to the manufacturer's protocol. Quantitative PCR was performed using SsoFast EvaGreen Supermixes (Bio‐Rad) and Bio‐Rad CFX™ Real Time system. The 2^−ΔΔCt^ method was used to quantify relative gene expression. Primer sequences are provided in Table S3.

### 2.8 Metabolic testing

For glucose and insulin tolerance tests (GTT, ITT), mice were fasted for 5 h in the morning. Baseline blood glucose was measured from a tail nick using a Precision Xceed Pro Glucometer, and then mice received an intraperitoneal injection of dextrose (2 g/kg, i.p., GTT) or insulin (1 U/kg, i.p., ITT), and then blood glucose was measured 15, 30, 60, 90, and 120 min after injection.

### 2.9 Statistical analysis

For cell culture experiments, individual data points represent the average value of all cells analyzed in a unique experimental replicate. Statistical analysis was performed using GraphPad Prism 9.4.0. Group differences were determined by Mann–Whitney tests, unpaired two‐tailed *t*‐tests or repeated measures two‐way ANOVA with LSD post hoc tests. Statistical significance was set at *p* < 0.05 and data are presented as mean ± SEM.

## 3 RESULTS

### 3.1 Aging increases endothelial cell telomere dysfunction

**FIGURE 1:**
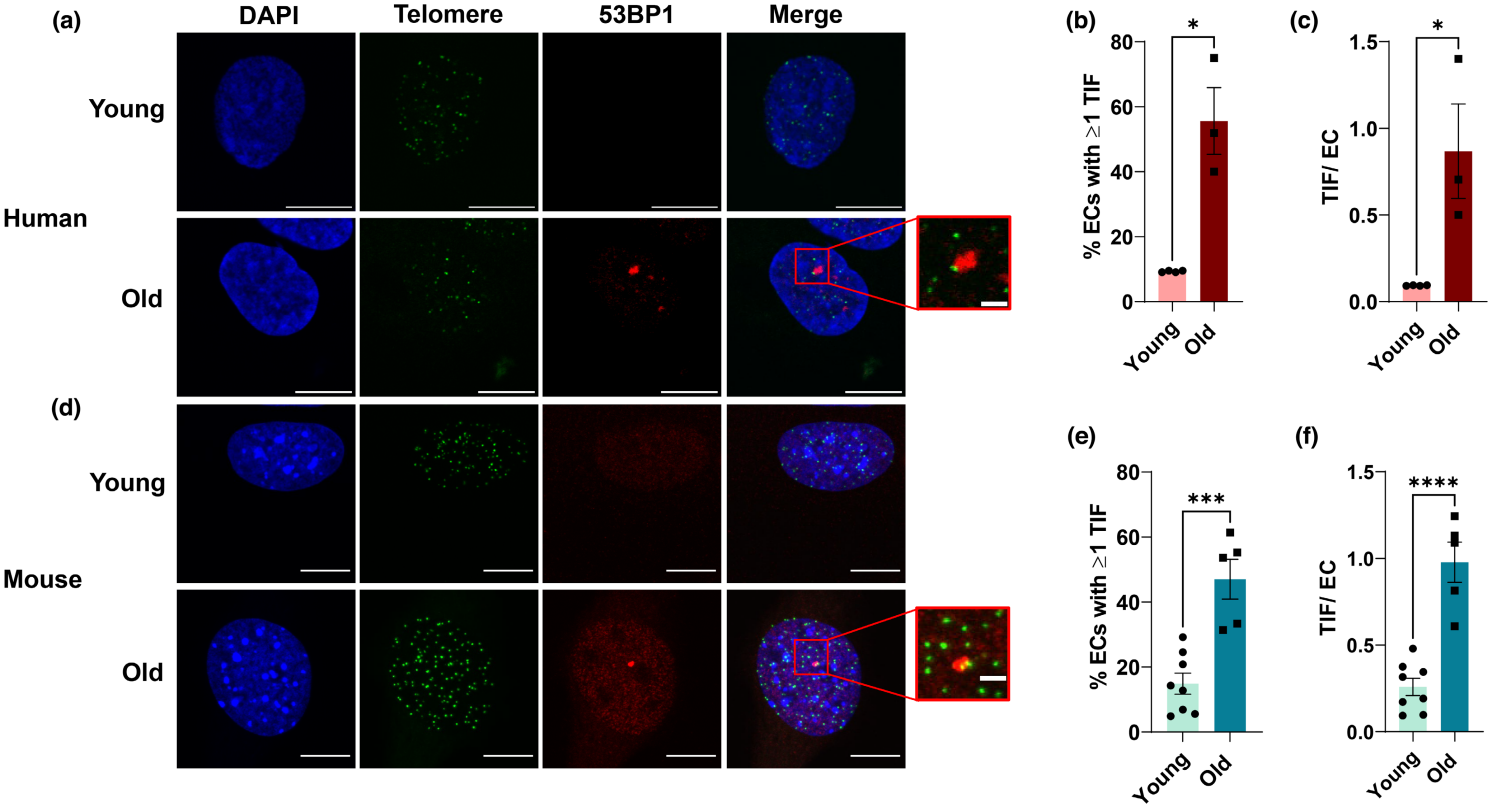
Effect of aging on lung endothelial cell (EC) telomere dysfunction. (a–c) Human lung endothelial cells from two young (29 ± 1 yo) and two old (67 ± 1 yo) donors. (a) Representative images of immunofluorescence fluorescent in situ hybridization performed on lung endothelial cells. (b) Percentage of endothelial cells containing one or more telomere dysfunction‐induced foci (TIF). *N* = 3–4 experimental replicates. (c) Number of telomere dysfunction‐induced foci (TIF) per endothelial cell. (d–f) Mouse lung endothelial cells from young (2.7 ± 0 mo) and old (27.3 ± 0 mo) mice. (d) Representative images of immunofluorescence fluorescent in situ hybridization performed on lung endothelial cells. (e) Percentage of endothelial cells containing one or more telomere dysfunction‐induced foci (TIF). (f) Number of telomere dysfunction‐induced foci (TIF) per endothelial cell. Scale bars 10 μm and 2 μm. *N* = 5–8 experimental replicates. Data are mean ± SEM. *p < 0.001; ****p < 0.0001.

To determine whether aging results in telomere dysfunction in microvascular endothelial cells similar to large arteries (Bloom, Tucker, et al., 2022), we purchased commercially available human lung microvascular endothelial cells from young and old human donors and performed IF‐FISH. The percentage of endothelial cells containing one or more TIF was elevated with aging (Figure 1A,B, *p* < 0.05) as well as the number of TIF per endothelial cell (Figure 1A,C, *p* < 0.05). To determine whether changes in length were responsible for increases in TIF, telomere length was ascertained from fluorescence intensity. Telomere length did not differ in endothelial cells of young and old donors (Figure S1A, *p* > 0.05) independent of whether cells did not (Figure S1B, *p* > 0.05) or did contain a TIF (Figure S1C, *p* > 0.05). Additionally, maximum and minimum telomere length were not different between young and old donors, independent of TIF status (Figure S1D, *p* > 0.05). To ascertain whether aging similarly impacted telomeres in mice, we isolated, cultured, and performed IF‐FISH on lung endothelial cells from young and old mice. Similar to humans, old mice displayed increases in the percentage of endothelial cells containing one or more TIF (Figure 1D,E, *p* < 0.001) and increased TIF per endothelial cell (Figure 1D,F, *p* < 0.0001). To determine whether changes in telomere length impacted the occurrence of TIF, we analyzed telomere length. In comparison with young mice, old mice had modestly longer telomeres (Figure S1E, *p* < 0.05) independent of whether the cells did not (Figure S1F, *p* > 0.05) or did contain a TIF (Figure S1G, *p* > 0.05). Neither maximum nor minimum telomere length was affected by age, regardless of TIF status, suggesting that increases in TIF occurred independently of critically short telomeres (Figure S1H, *p* > 0.05). Taken together, these data suggest that aging results in endothelial cell telomere dysfunction independent of length in both humans and mice.

### 3.2 Reduced endothelial cell *Trf2* increases telomere dysfunction and senescence and impairs endothelial function

**FIGURE 2:**
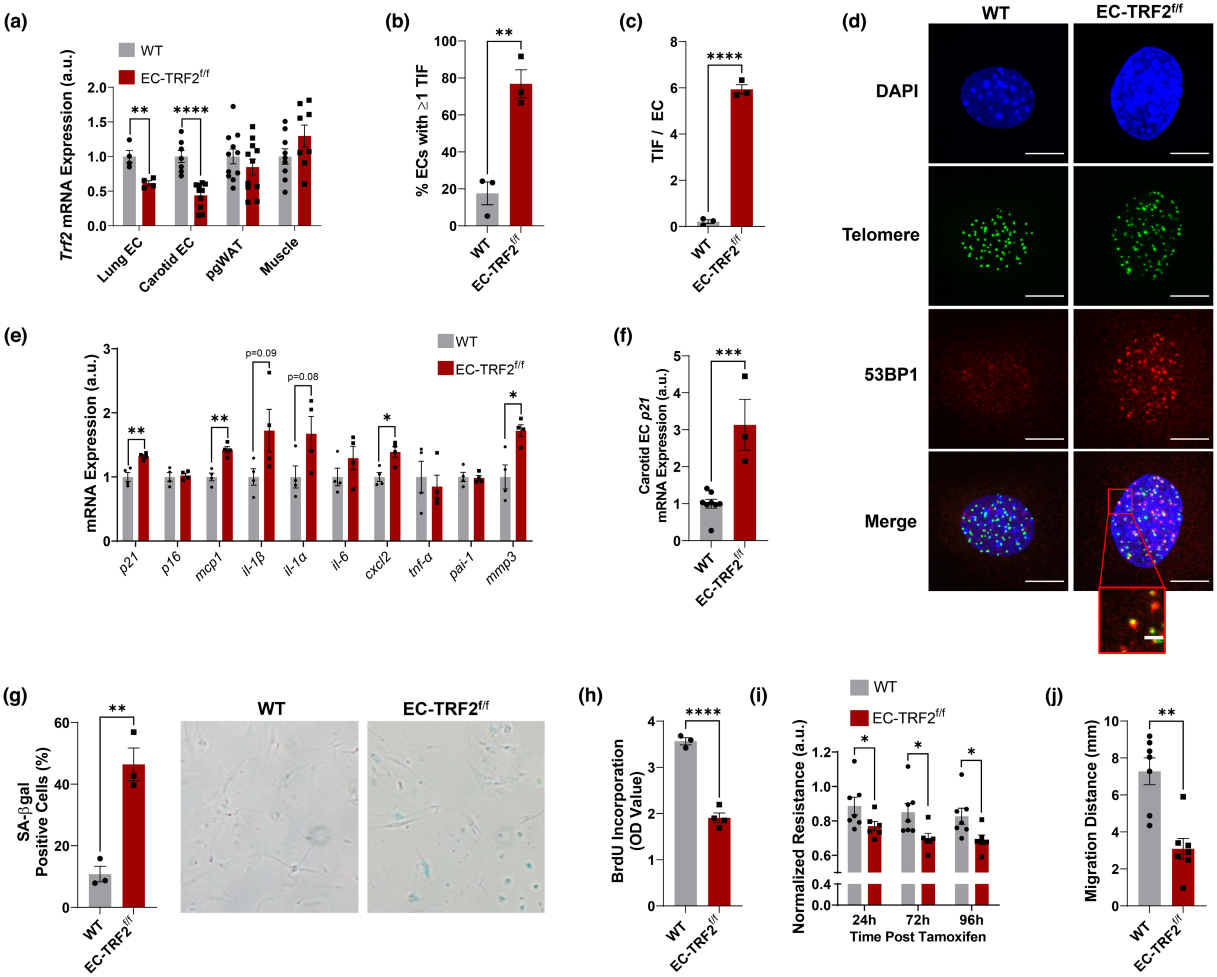
Effect of endothelial cell (EC) *Trf2* reduction on telomere dysfunction, senescence, and endothelial function. (a) *Trf2* mRNA expression in lung endothelial cells, carotid artery endothelial cells, perigonadal white adipose tissue (pgWAT), and skeletal muscle. (b) Percentage of endothelial cells containing one or more telomere dysfunction‐induced foci (TIF). (c) Number of telomere dysfunction‐induced foci (TIF) per endothelial cell. (d) Representative images of immunofluorescence fluorescent in situ hybridization performed on lung endothelial cells from WT and EC‐TRF2^f/f^ mice. (e) Lung endothelial cell mRNA expression for senescence markers and SASP factors. (f) Carotid artery endothelial cell *p21* mRNA expression. (g) Percentage of lung endothelial cells expressing senescence‐associated β‐galactosidase (SA‐βgal). (h) BrdU incorporation in lung endothelial cells. (i) Normalized transendothelial resistance, a measure of barrier function, of lung endothelial cells detected using electric cell‐substrate impedance sensing. (j) Lung endothelial cell migration distance from scratch migration assay. Scale bars 10 μm and 2 μm. *N* = 3–11 per group. Data are mean ± SEM. *p < 0.05; **p < 0.01; ***p < 0.001; ****p < 0.0001.

To examine whether telomere dysfunction is capable of inducing endothelial cell senescence, we generated a mouse model allowing for selective reduction of the telomere shelterin protein TRF2, by crossing mice containing Lox‐P sites flanking Exons 1 and 2 of the *Trf2* gene to mice containing a tamoxifen‐inducible endothelial cell‐specific Cre‐recombinase (Cdh5‐CreERT2). Mice containing Cre but lacking Lox‐P sites (WT) were compared to mice containing both Cre and Lox‐P sites (EC‐TRF2^f/f^). For all experiments, both WT and EC‐TRF2^f/f^ mice received tamoxifen, and male and female ~3.5‐mo‐old littermates were evaluated in approximately equal distributions (Table S1). To validate our model, we examined mRNA expression of *Trf2* in lung endothelial cells, excised carotid arteries perfused with a lysis reagent that allows us to collect an endothelial cell‐enriched effluent, as well as perigonadal white adipose tissue (pgWAT) and skeletal muscle. In comparison with WT mice, both lung (*p* < 0.01) and carotid artery endothelial cells (*p* < 0.0001) from EC‐TRF2^f/f^ mice displayed approximately a 50% reduction in *Trf2* mRNA expression, whereas pgWAT and muscle *Trf2* mRNA expression was not different (Figure 2A, *p* > 0.05). EC‐TRF2^f/f^ mice tended to have greater body and pgWAT mass, and had significantly elevated kidney mass, although kidney mass was no longer different when normalized to body mass (Table S1).

To evaluate whether reductions in *Trf2* expression resulted in telomere dysfunction, we isolated lung endothelial cells from WT and EC‐TRF2^f/f^ mice and performed IF‐FISH. The percentage of endothelial cells from EC‐TRF2^f/f^ mice with one or more TIF was greater than in WT mice (Figure 2B,D, *p* < 0.01) as was the number of TIF per endothelial cell (Figure 2C,D, *p* < 0.0001). Changes in telomere length were not responsible for increases in TIF in EC‐TRF2^f/f^ mice as mean telomere length was not different between groups (Figure S2A, *p* > 0.05) even when stratified by TIF status (Figure S2B,C, *p* > 0.05). Furthermore, in EC‐TRF2^f/f^ mice, maximum telomere length was greater in cells that did not contain TIF; however, there were no differences in either maximum telomere length in cells containing TIF or in minimum telomere length independent of TIF status (Figure S2D, *p* > 0.05).

To determine whether increases in TIF resulted in senescence, we examined mRNA expression for senescence markers and SASP factors. EC‐TRF2^f/f^ mice displayed increases in *p21* mRNA expression in lung endothelial cells (Figure 2E, *p* < 0.001) and carotid artery endothelial cell‐enriched effluents (Figure 2F, *p* < 0.01), and increases in lung endothelial cell expression of SASP factors *mcp1*, *cxcl2*, and *mmp3* (Figure 2E, *p* < 0.05). Next, we examined the percentage of senescence‐associated β‐galactosidase (SA‐βgal) cells and BrdU incorporation, a marker of cell division. As would be expected with induction of senescence, there were more SA‐βgal positive cells in endothelial cells from EC‐TRF2^f/f^ mice (Figure 2G, *p* < 0.01) as well as reduced BrdU incorporation (Figure 2H, *p* < 0.0001) compared to their WT counterparts. Additionally, endothelial cells from EC‐TRF2^f/f^ mice exhibited impairments in barrier function (Figure 2I, *p* < 0.05) and cell migration (Figure 2J, *p* < 0.01). Taken together, these data suggest that the reduction in endothelial cell *Trf2* expression results in telomere dysfunction‐induced senescence that impairs endothelial cell function.

### 3.3 Endothelial cell telomere dysfunction impairs endothelium‐dependent dilation due to increases in reactive oxygen species

**FIGURE 3:**
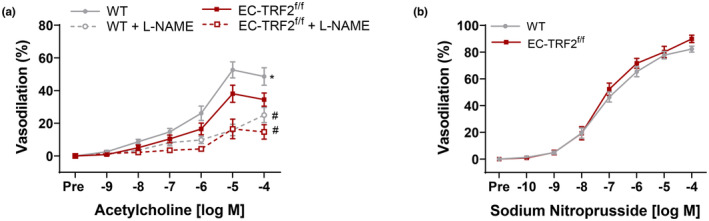
Effect of telomere dysfunction‐induced senescence on endothelium‐dependent and ‐independent vasodilation. (a) Dose–response curves to acetylcholine in mesenteric arteries in the absence and presence of the nitric oxide synthase inhibitor l‐NAME. (b) Dose–response curves to the exogenous nitric oxide donor sodium nitroprusside in mesenteric arteries. *N* = 13–17 per group. Data are mean ± SEM. *p < 0.05 versus EC‐TRF2^f/f^ acetylcholine, ^#^ *p* < 0.05 versus acetylcholine without l‐NAME.

To examine the physiological consequences of telomere dysfunction‐induced senescence, we excised and cannulated mesenteric arteries and examined vasodilation to ACh. In comparison with WT controls, EC‐TRF2^f/f^ mice displayed reduced ACh‐mediated vasodilation (Figure 3A, *p* < 0.05). To determine whether alterations in the bioavailability of NO might be responsible for impairments in dilation in EC‐TRF2^f/f^ mice, responses to ACh were repeated in the presence of the NO synthase inhibitor l‐NAME. In the presence of l‐NAME, vasodilation was reduced in both groups. However, l‐NAME abolished differences between groups suggesting reduced NO bioavailability in EC‐TRF2^f/f^ mice (Figure 3A, *p* > 0.05). Changes in vasodilatory capacity seen in EC‐TRF2^f/f^ mice occurred independently of smooth muscle responsiveness to NO, evidenced by similar vasodilatory responses to the exogenous NO donor SNP (Figure 3B, *p* > 0.05). Furthermore, sensitivity to vasodilators was not different between groups, evidenced by similar half maximal effective concentrations (EC_50_, Table S2, *p* > 0.05). These data demonstrate that telomere dysfunction‐induced senescence impairs EDD.

**FIGURE 4:**
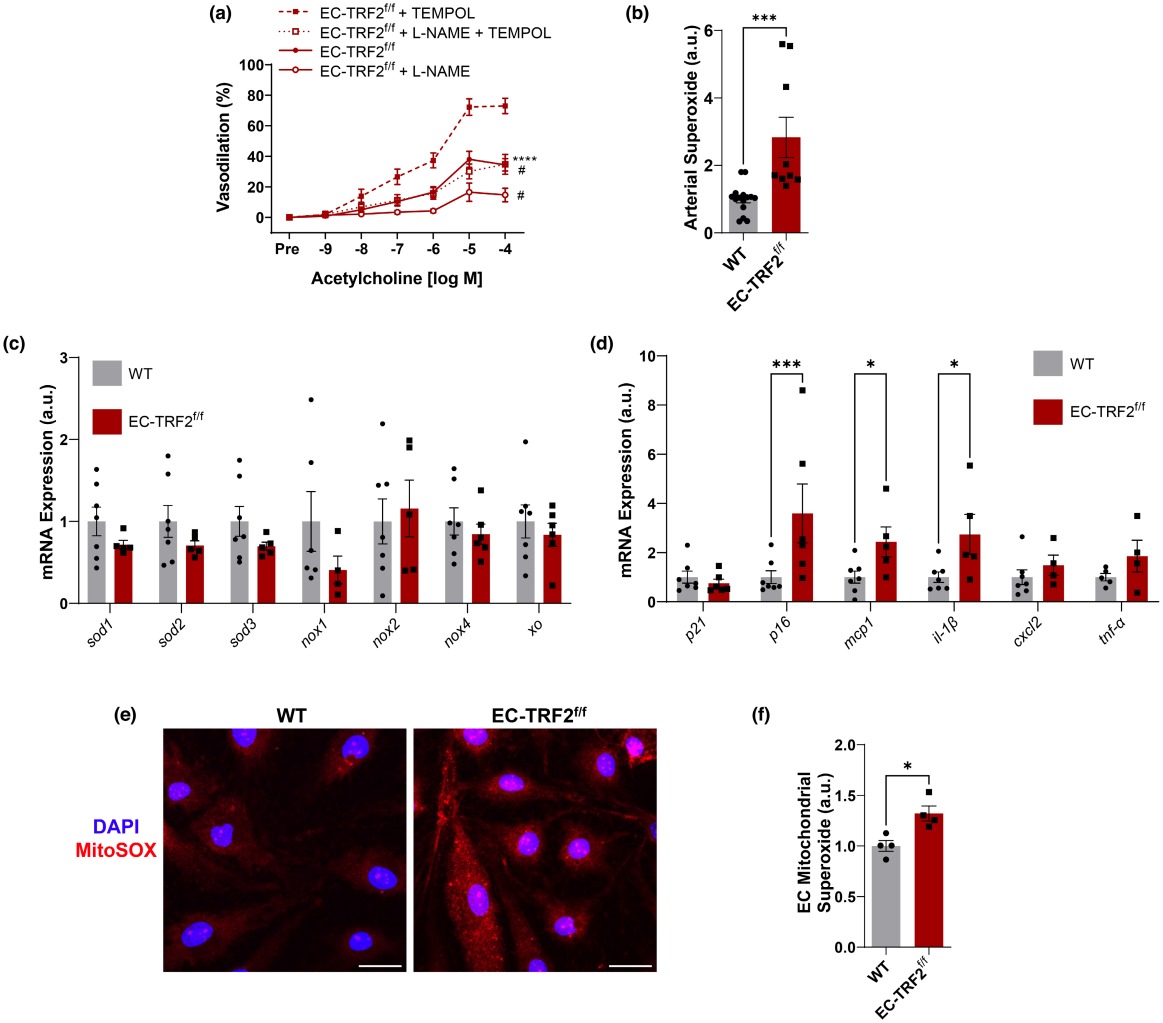
Effect of telomere dysfunction‐induced senescence on oxidative stress and inflammation. (a) Dose–response curves to acetylcholine in mesenteric arteries in the absence and presence of the nitric oxide synthase inhibitor l‐NAME and the superoxide scavenger TEMPOL. *N* = 7–13 per group. ****p<0.0001 versus acetylcholine with TEMPOL; ^#^ *p* < 0.05 versus acetylcholine without l‐NAME. (b) Electron paramagnetic resonance spectrometry using CMH spin probe to assess superoxide in carotid artery segments. *N* = 9–15 per group. (c) Whole mesenteric artery lysate mRNA expression of antioxidant and oxidant enzyme genes. Each dot represents pooled arteries from two mice. *N* = 4–7 per group. (d) Whole mesenteric artery lysate mRNA expression of senescence markers and SASP factors. Each dot represents pooled arteries from two mice. *N* = 4–7 per group. (e) Representative images of lung endothelial cells stained with MitoSOX to examine mitochondrial superoxide. Scale bars 10 μm. (f) Quantification of lung endothelial cells stained with MitoSOX to examine mitochondrial superoxide. *N* = 4 per group. Data are mean ± SEM. *p < 0.05; ***p < 0.001; ****p < 0.0001.

To determine whether telomere dysfunction‐induced senescence reduces EDD due to elevations in ROS, we repeated responses to ACh in vessels preincubated with the superoxide scavenger TEMPOL. In comparison with ACh alone, TEMPOL improved ACh‐mediated EDD in EC‐TRF2^f/f^ mice in both the absence and presence of L‐NAME (Figure 4A, *p* < 0.0001). Electron paramagnetic resonance spectrometry confirmed that EC‐TRF2^f/f^ mice produced more arterial superoxide compared to their WT counterparts (Figure 4B, *p* < 0.001). To examine whether reductions in antioxidant expression or increases in expression of oxidant‐producing enzymes might be responsible for increased ROS, we evaluated gene expression of isoforms of superoxide dismutase, NADPH oxidases, and xanthine oxidase in whole mesenteric artery lysates. Gene expression for these mediators of arterial ROS was not different between groups (Figure 4C, *p* > 0.05); however, expression of the senescence marker *p16* and SASP factors *mcp1* and *il‐1β* was elevated (Figure 4D, *p* < 0.05). To evaluate the contribution of mitochondrial derived ROS, we isolated lung endothelial cells and examined mitochondrial ROS bioactivity with the fluorogenic stain MitoSOX. Endothelial cells from EC‐TRF2^f/f^ had elevated mitochondrial superoxide levels in comparison with their WT counterparts (Figure 4E,F, *p* < 0.05). Taken together, these data demonstrate that telomere dysfunction‐induced senescence impairs EDD due to increases in ROS that are at least in part driven by mitochondrial superoxide levels.

### 3.4 Endothelial cell telomere dysfunction induces senescence and inflammatory gene expression in metabolically active tissues and impairs metabolic function

**FIGURE 5:**
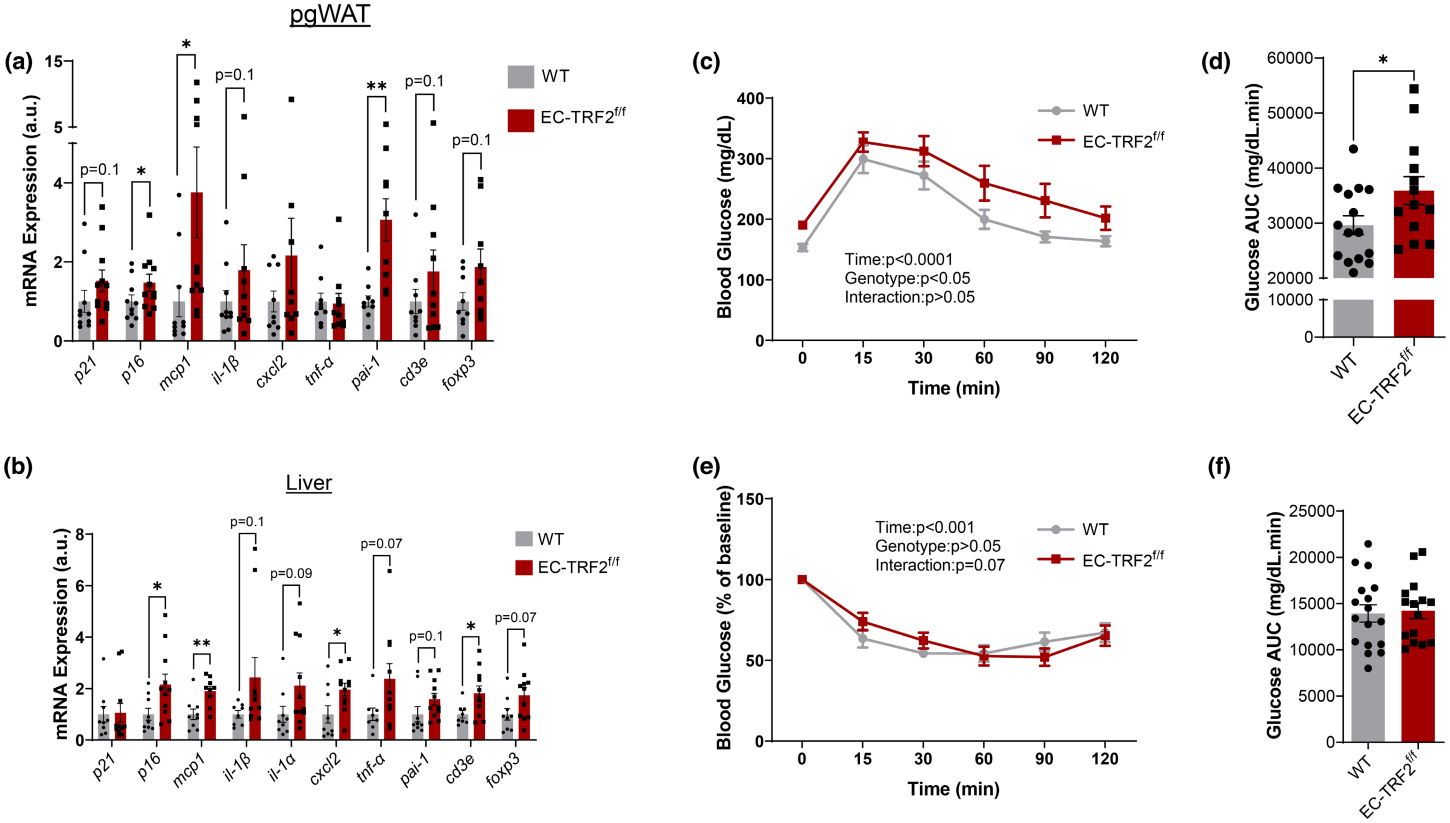
Effect of telomere dysfunction‐induced senescence and SASP expression in metabolically relevant tissues and metabolic function. (a) mRNA expression of senescence and SASP markers in perigonadal white adipose tissue (pgWAT). (b) mRNA expression of senescence and SASP markers in liver. (c) Blood glucose response curve during glucose tolerance test. (d) Area under the curve (AUC) during a glucose tolerance test. (e) Blood glucose response curve during insulin tolerance test. (f) Area under the curve (AUC) during an insulin tolerance test. *N* = 8–15 per group. Data are mean ± SEM. *p < 0.05; **p < 0.01.

To explore a putative role of endothelial cell senescence in impacting metabolically active tissues, we isolated mRNA from pgWAT and the liver and examined markers of senescence and the SASP. In pgWAT, EC‐TRF2^f/f^ mice displayed increased expression of the senescence marker *p16* alongside SASP factors *mcp1* and *pai‐1* (Figure 5A, *p* < 0.05). In the liver, EC‐TRF2^f/f^ mice similarly displayed elevated expression of *p16* and *mcp1*, as well as elevated *cxcl2* and *cd3e* (Figure 5B, *p* < 0.05). Because adipose tissue and the liver are important in the maintenance of glucose homeostasis, we examined whether these alterations in senescence and inflammation resulted in functional consequences. Compared to WT mice, EC‐TRF2^f/f^ displayed elevated blood glucose curves (Figure 5C, *p* < 0.05) and area under the curve (Figure 5D, *p* < 0.05) during an intraperitoneal glucose tolerance test. Insulin tolerance tended to be different between WT and EC‐TRF2^f/f^ mice (Figure 5E, *p* = 0.07). These data demonstrate that telomere dysfunction in endothelial cells induces senescence and increases inflammation within several metabolically active tissues thereby impacting systemic glucose metabolism.

### 3.5 Endothelial cell telomere dysfunction impairs insulin‐stimulated endothelium‐dependent dilation and reduces perfused microvascular density

**FIGURE 6:**
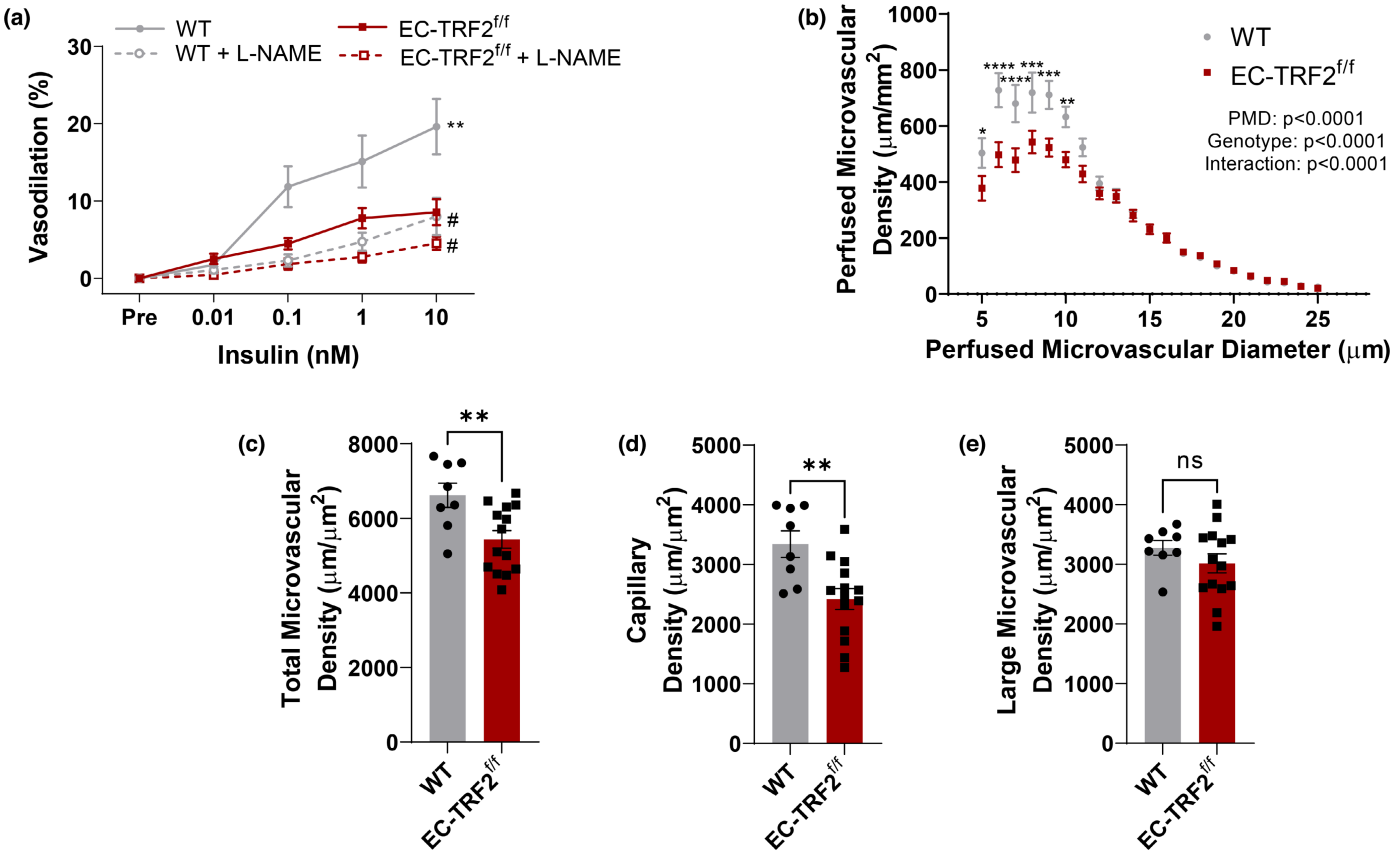
Effect of telomere dysfunction‐induced senescence on endothelium‐dependent to insulin and perfused microvascular density. (a) Dose–response curves to insulin in mesenteric arteries in the absence and presence of the nitric oxide synthase inhibitor l‐NAME. (b) Perfused microvascular density in 5–25 μm microvessels. (c) Perfused microvascular density in 5–25 μm microvessels. (d) Perfused microvascular density in 5–9 μm microvessels. (e) Perfused microvascular density in 10–25 μm microvessels. *N* = 8–14 per group. Data are mean ± SEM. *p < 0.05; **p < 0.01.

In addition to the altered inflammatory profile of metabolically active tissues in EC‐TRF2^f/f^ mice, we examined whether alterations in systemic glucose metabolism might be driven through vascular phenotypes that would influence nutrient delivery to metabolically active tissues. Insulin‐mediated EDD was reduced in mesenteric arteries of EC‐TRF2^f/f^ mice compared with WT controls (Figure 6A, *p* < 0.01). l‐NAME reduced dilation in both groups (Figure 6A, *p* < 0.05); however, dilation to insulin in the presence of l‐NAME was not different between groups (Figure 6A, *p* > 0.05). The density of perfused microvessels was reduced in the mesentery of EC‐TRF2^f/f^ mice (Figure 6B,C, *p* < 0.01), due to reductions in perfused capillary density (Figure 6D, *p* < 0.01) but not larger microvessels between 10 and 25 μm in size (Figure 6E, *p* > 0.05). These observations demonstrate that endothelial cell telomere dysfunction impairs insulin‐stimulated EDD and reduces the number of perfused microvessels which may contribute to altered metabolic function.

## 4 DISCUSSION

The key novel findings of the present study are as follows. First, we found that aging increases the abundance of TIF in endothelial cells independent of changes in telomere length. Second, we found that inducing telomere dysfunction in endothelial cells results in senescence and increases the expression of a subset of SASP factors. Third, telomere dysfunction‐induced senescence impairs endothelial function, including cell migration and barrier function, as well as EDD due to increases in mitochondrial ROS. Finally, we demonstrated that telomere dysfunction‐induced senescence in endothelial cells results in senescence marker and SASP factor expression in adipose tissue and the liver and reduces insulin‐stimulated EDD and perfused microvascular density, which collectively contribute to the observed impairments in metabolic function.

### 4.1 Aging results in endothelial cell telomere dysfunction

The mechanisms responsible for the induction of endothelial cell senescence with advancing age remain incompletely understood. Telomeres act as sensors of both replicative and oxidative stress, and telomere dysfunction is persistent and resists repair resulting in sustained activation of the DDR (Barnes et al., 2022; Fumagalli et al., 2012; Hewitt et al., 2012; Oikawa & Kawanishi, 1999; Opresko et al., 2005; Victorelli & Passos, 2017). Accordingly, TIF are highly predictive of senescence induction and considered high‐fidelity markers of senescence (Fumagalli et al., 2012; González‐Gualda et al., 2021; Hewitt et al., 2012; Rossiello et al., 2022; Uryga et al., 2021). Here, we demonstrate that aging increases the amount of lung endothelial cells containing dysfunctional telomeres, as well as the number of TIF per endothelial cell, demonstrating that telomere dysfunction occurs in microvascular endothelial cells similar to the aorta (Bloom, Tucker, et al., 2022) and is seen in both humans and mice. Similar to mouse aorta (Bloom, Tucker, et al., 2022) and human arteries (Morgan et al., 2013), we found that the increased abundance of TIF occurs independently of changes in telomere length. These studies indicate that endothelial telomere dysfunction likely occurs independently of cell replication that could lead to critically short telomeres, in line with evidence that endothelial cells are one of the slowest replicating cell types (Jones et al., 2022) that are not postmitotic. Surprisingly, in lung endothelial cells from old mice, telomere length was greater than in young mice. This could be a consequence of telomerase (the enzyme that elongates telomeres) activity, which is retained in some mouse tissues (Prowse & Greider, 1995), or due to increased replication of young cells compared to old during their brief time in culture prior to analysis. Importantly, because aging did not result in critically short telomeres, our findings suggest that telomere dysfunction in the endothelium may be a result of oxidative damage at telomeres (Barnes et al., 2022; Liu et al., 2019; Opresko et al., 2005) from endothelial‐derived oxidative stress or exposure to high partial pressures of oxygen and oxidants in the circulation. In line with this logic, oxidative stress can accelerate endothelial cell senescence by impacting telomeres (Kurz et al., 2004). Moreover, evidence suggests that lifestyle factors that increase oxidative stress, such as obesity, may influence telomere regulation (Bloom et al., 2020) and senescence in endothelial cells (Hasegawa et al., 2012), thereby accelerating biological aging.

### 4.2 Induction of telomere dysfunction in endothelial cells results in senescence

Identifying the inducer of senescence holds important implications because with advancing age endothelial cells are one of the first cell types to become senescent, for example, as is seen in liver sinusoidal endothelial cells (Grosse et al., 2020). Moreover, tissues with a high abundance of endothelial cells have the highest senescence burden (Yousefzadeh et al., 2020), and senescence in endothelial cells may have wide‐ranging physiological consequences (Bloom, Islam, et al., 2022). Here, we demonstrate that endothelial‐specific reduction of the telomere shelterin gene, *Trf2*, results in an increase in the abundance of TIF that subsequently induces senescence. These findings corroborate prior work demonstrating that telomere dysfunction in human and mouse arteries is associated with senescence (Cardus et al., 2013; Morgan et al., 2013; Morgan et al., 2019; Uryga et al., 2021). Taken together, results from the present study are in agreement with previous findings and also provide additional evidence that telomere dysfunction is an upstream cause of endothelial senescence.

### 4.3 Consequences of telomere dysfunction‐induced senescence

The vascular endothelium is a highly dynamic monolayer of cells that lines the vascular network and plays important roles in maintaining systemic physiological function (Bloom, Islam, et al., 2022). With advancing age, endothelial function, which can be assessed via EDD, is impaired and contributes to the development of CVD (Donato et al., 2018). Evidence exists supporting an association between endothelial senescence and age‐related impairments in EDD (Liu et al., 2019; Roos et al., 2016; Rossman et al., 2017); however, direct cause and effect evidence of this relationship is limited (Bautista‐Niño et al., 2020; Bloom, Islam, et al., 2022). In the present study, we demonstrate that endothelial telomere dysfunction‐induced senescence impairs EDD. Similarly, other cell phenotypes associated with impaired endothelial function including cell migration and barrier function were adversely impacted. These findings are important because they link an inducer of senescence (telomere dysfunction) that occurs with advancing age to functional consequences that bear clinical implications and therefore may represent one of the initial precipitating events that leads to the development of CVD.

In advanced age, elevations in oxidative stress and inflammation impair EDD by reducing the bioavailability of NO (Donato et al., 2018). In the present study, telomere dysfunction‐induced senescence increased endothelial cell expression levels of several SASP factors and increased levels of superoxide. This resulted in impaired EDD evidenced by restored vasodilation following preincubation of vessels with the superoxide scavenger TEMPOL. These data support the idea that an increased abundance of senescent endothelial cells contributes directly to impaired EDD through elevations in ROS (Bloom, Islam, et al., 2022; Liu et al., 2019). The source of ROS in senescent endothelial cells remains elusive. Examination of arterial expression of antioxidant and pro‐oxidant enzymes was not different between groups, suggesting that these were not the source of ROS. Evidence suggests that mitochondrial ROS is elevated in senescent endothelial cells (Lafargue et al., 2017), a feature that is necessary to enforce senescence in fibroblasts (Passos et al., 2010). Likewise, in the present study, telomere dysfunction‐induced senescence increases endothelial cell levels of mitochondrial superoxide, which could potentially form a positive feedback loop and further damage telomeres as is seen in cardiomyocytes (Anderson et al., 2019). However, future research on this relationship between ROS and endothelial cell telomere dysfunction is warranted. Cumulatively, the findings of the present study identify telomere dysfunction as a mechanism that induces senescence and subsequently inflammation and oxidative stress that impair endothelial function.

Through control of vasodilation, as well as through the formation of new blood vessels, the endothelium regulates blood flow distribution and thereby is capable of augmenting nutrient delivery to meet the demand of metabolically active tissues. Additionally, endothelial cells reside within every tissue; therefore, SASP factors from senescent endothelial cells can act as a local source of inflammation that impairs tissue function or potentially induce senescence in neighboring cells, known as a bystander effect (Nelson et al., 2012). To explore this hypothesis, we evaluated expression levels of senescence markers and SASP factors within several tissues. In whole artery lysates comprised largely of vascular smooth muscle cells, gene expression of senescence and SASP markers were elevated. These findings are in agreement with previous studies which show that SASP from endothelial cells increases inflammatory gene expression in vascular smooth muscle cells (van der Feen et al., 2020), and provide novel evidence that the endothelial SASP may also induce vascular smooth muscle cell senescence.

Similar to whole artery lysates, we found increased gene expression of senescence and SASP markers in the liver and adipose tissue. Because the liver and adipose tissue are important for the maintenance of metabolic homeostasis, we wanted to determine whether the altered inflammatory state of these tissues resulted in physiological consequences. Indeed, endothelial telomere dysfunction‐induced senescence impaired glucose tolerance while insulin tolerance tended to be affected. Moreover, EC‐TRF2^f/f^ mice tended to have greater body and adipose tissue mass, and had greater kidney mass, suggesting wide‐ranging systemic effects of endothelial telomere dysfunction‐induced senescence that warrants future investigations. In addition to the altered inflammatory state of liver and adipose tissue, and changes in body and tissue mass, we explored whether dilation in response to metabolically relevant dilators or reduced perfusion of tissues might contribute to alterations in metabolic homeostasis. Interestingly, endothelial telomere dysfunction‐induced senescence reduced vasodilation to insulin and reduced the perfused microvascular density due to a reduction in the number of perfused capillaries. Such alterations in vascular function may contribute to impairments in metabolic function by limiting the delivery of glucose and insulin to metabolically active tissues. These findings are congruent with prior research that demonstrates that overexpression of a dominant negative TRF2 in endothelial cells induces senescence in adipose tissue and disrupts metabolic homeostasis (Barinda et al., 2020), and conversely, endothelial‐specific deletion of p53 attenuates high‐fat diet‐induced obesity and the accompanying metabolic impairments (Yokoyama et al., 2014). Importantly, this area of research highlights endothelial senescence as a potential therapeutic target for age‐ and obesity‐induced metabolic dysfunction.

## 5 CONCLUSIONS

In the present study, we demonstrated that advanced age resulted in an increased abundance of dysfunctional telomeres. Using an endothelial‐specific model to induce telomere dysfunction, we found that telomere dysfunction induces senescence resulting in a SASP characterized by elevated inflammation and oxidative stress that impair endothelial function. Furthermore, we found that endothelial cell telomere dysfunction‐induced senescence impaired vasodilation to insulin, reduced perfused capillary density, and increased inflammation in metabolically active tissues which resulted in metabolic dysfunction. These findings provide important insight into a contributing factor that increases oxidative stress and inflammation known to impair endothelial function by identifying telomere dysfunction‐induced senescence as a source, and enhance our understanding of the consequences of endothelial cell senescence.

## AUTHOR CONTRIBUTIONS

SIB, YL, and AJD designed the study. SIB, YL, JRT, MTI, DRM, HA, TGT, RCB, LAL, and AJD performed the experiments. SIB, JRT, HA, and TGT analyzed the data. SIB and AJD interpreted the data and wrote the manuscript. All authors read and approved the final version of the manuscript.

## CONFLICT OF INTEREST STATEMENT

A.J.D. is a scientific adviser and stockholder and L.A.L. is a stockholder in Recursion Pharmaceuticals. None of the work done with Recursion is outlined or discussed in this manuscript. The other authors declare no competing interests.

## Supporting information


**Data S1:** Supporting Information

## Data Availability

The data that support the findings of this study are available from the corresponding author upon reasonable request.
