# Supplementary material for: Endothelial cell telomere dysfunction induces senescence and results in vascular and metabolic impairments
Source: Aging Cell. 2023 May 31;22(8):e13875. doi: 10.1111/acel.13875 (PMC10410008; doi:10.1111/acel.13875)
Supplement: Supplementary file 1 — Data S1: Supporting Information [file ACEL-22-e13875-s001.pdf]

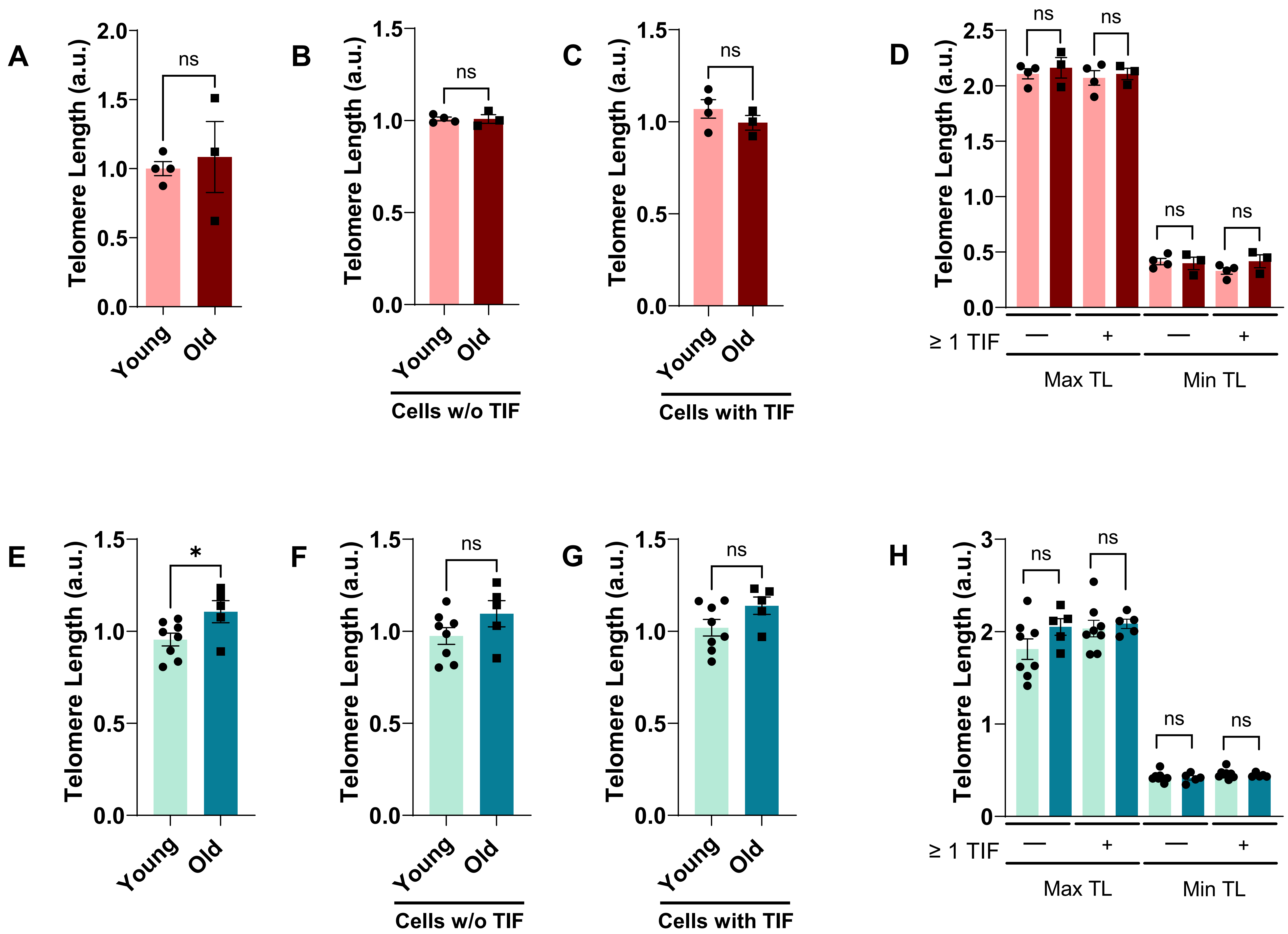

Supplementary Figure 1. Effect of aging on lung endothelial cell telomere length and influence of telomere length on telomere dysfunction. (A-D) Human lung endothelial cell telomere analysis from two young (29 ± 1 yo) and two old (67 ± 1 yo) donors. (A) Mean telomere length in endothelial cells. (B) Mean telomere length in cells without (w/o) one or more telomere dysfunction-induced foci (TIF). (C) Mean telomere length in cells containing one or more telomere dysfunction-induced foci (TIF). (D) Maximum and minimum telomere length in endothelial cells with and without one or more telomere dysfunction-induced foci (TIF). N = 3 – 4 experimental replicates. (E-H) Mouse lung endothelial cells. (E) Mean telomere length in endothelial cells. (F) Mean telomere length in cells without (w/o) one or more telomere dysfunction-induced foci (TIF). (G) Mean telomere length in cells containing one or more telomere dysfunction-induced foci (TIF). (H) Maximum and minimum telomere length in endothelial cells with and without one or more telomere dysfunction-induced foci (TIF). N = 5 – 8 experimental replicates. Data are mean ± SEM. *$p < 0.05$.

Table S1. Animal characteristics

Genotype	WT	EC-TRF2$^{f/f}$	*p*
N (male/female)	25 (13/12)	23 (12/11)	0.99
Age (mo)	3.5 ± 0.1	3.6 ± 0.2	0.51
Body Mass (g)	21.9 ± 0.5	23.4 ± 0.7	0.09
Heart Mass (mg)	117.9 ± 6.5	126.0 ± 6.7	0.39
Heart: Body Mass (mg/g)	5.6 ± 0.3	5.4 ± 0.3	0.71
pgWAT (mg)	247.0 ± 21.9	315.7 ± 34.1	0.09
pgWAT: Body Mass (mg/g)	11.5 ± 1.0	14.0 ± 1.5	0.15
Liver (mg)	1176.4 ± 66.6	1312.0 ± 56.2	0.13
Liver: Body Mass (mg/g)	55.0 ± 2.9	57.2 ± 1.5	0.52
Kidney (mg)	276.9 ± 14.8	316.8 ± 10.0	0.03
Kidney: Body Mass (mg/g)	13.2 ± 0.8	13.7 ± 0.3	0.56
Spleen (mg)	116.2 ± 15.2	102.3 ± 5.8	0.37
Speen: Body Mass (mg/g)	4.9 ± 0.7	4.5 ± 0.3	0.53
Quad (mg)	298.3 ± 15.7	325.7 ± 17.0	0.25
Quad: Body Mass (mg/g)	13.9 ± 0.7	14.4 ± 0.7	0.62
Soleus (mg)	10.8 ± 2.2	11.9 ± 1.6	0.67
Soleus: Body Mass (mg/g)	0.5 ± 0.1	0.4 ± 0.1	0.81

Data are mean ± SEM

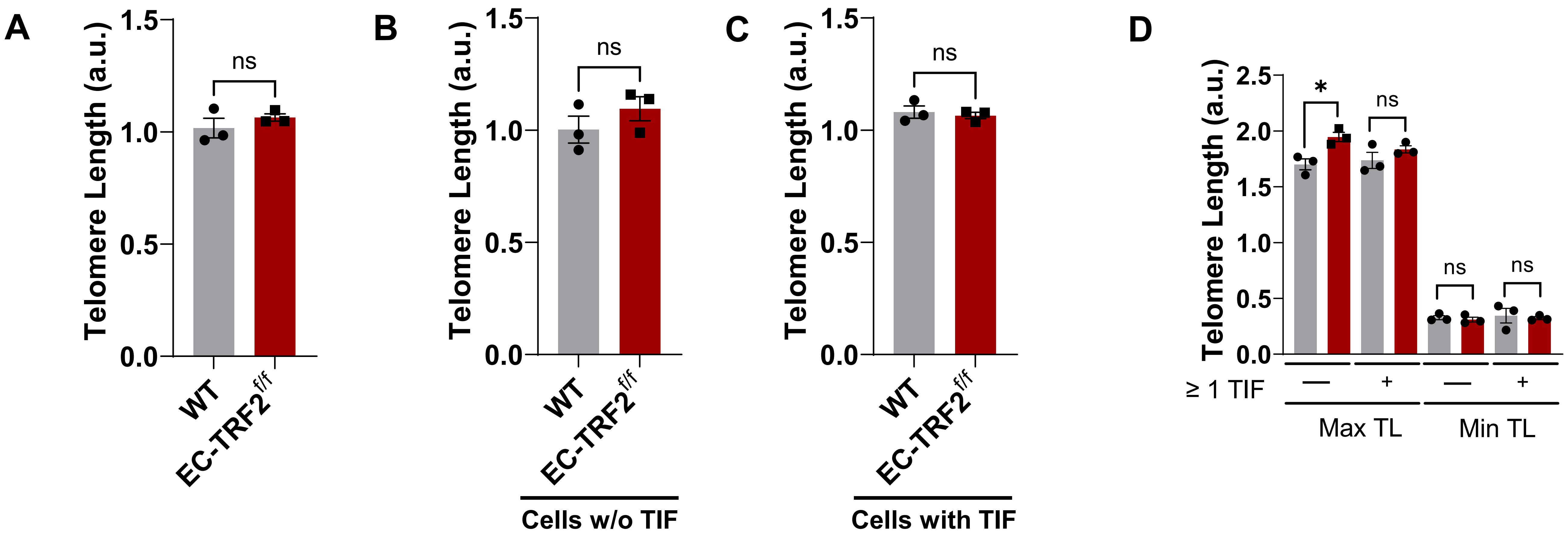

Supplementary Figure 2. Effect of endothelial cell *Trf2* reduction on telomere length, and influence of telomere length on telomere dysfunction. (A) Mean telomere length in endothelial cells. (B) Mean telomere length in cells without (w/o) one or more telomere dysfunction-induced foci (TIF). (C) Mean telomere length in cells containing one or more telomere dysfunction-induced foci (TIF). (D) Maximum and minimum telomere length in endothelial cells with and without one or more telomere dysfunction-induced foci (TIF). N = 3 per group. Data are mean ± SEM. *$p < 0.05$.

Table S2. EC_{50} of mesenteric artery dose-response curves

**Genotype**	**WT**	**EC-TRF2$^{f/f}$**	***p***
EC_{50}, log *M*			
ACh	-6.0 ± 0.2	-6.0 ± 0.2	0.33
ACh + L-NAME	-5.4 ± 0.3	-5.2 ± 0.2	0.55
SNP	-7.3 ± 0.1	-7.2 ± 0.2	0.55
ACh + TEMPOL	-6.6 ± 0.3	-6.2 ± 0.3	0.99
ACh + TEMPOL + L-NAME	-6.6 ± 0.4	-6.3 ± 0.7	0.39
EC_{50}, nM			
Ins	0.8 ± 0.4	0.3 ± 0.2	0.20
Ins + L-NAME	1.5 ± 0.6	1.2 ± 0.5	0.76

Data are mean ± SEM

Table S3. Primer sequences for RT-qPCR

Genes	Forward	Reverse
18s	5′-TAGAGGGACAAGTGGCGTTC-3′	5′-CGCTGAGCCAGTCAGTGT-3′
rplp0	5′-AGATTCGGGATAGCTGTTGGC-3′	5′-TCGGGTCCTAGACCAGTGTTC-3′
Trf2	5′-GTGGAACAGCCCTAACGGG-3′	5′-CCACTCGCTTTCTTCTATGGTC-3′
p16	5′-CGCAGGTTCTTGGTCACTGT-3′	5′-TGTTCACGAAAGCCAGAGCG-3′
p21	5′-CCTGGTGATGTCCGACCTG-3′	5′-CCATGAGCGCATCGCAATC-3′
mcp1	5′-GCATCCACGTGTTGGCTCA-3′	5′-CTCCAGCCTACTCATTGGGATCA-3′
il-1β	5′-CACAGCAGCACATCAACAAG-3′	5′-GTGCTCATGTCCTCATCCTG-3′
il-1α	5′-CGAAGACTACAGTTCTGCCATT-3′	5′-GACGTTTCAGAGGTTCTCAGAG-3′
il-6	5′-CTGGGAAATCGTGGAAT-3′	5′-CCAGTTTGGTAGCATCCATC-3′
cxcl2	5′- CCTGGTTCAGAAAATCATCCA-3′	5′-CTTCCGTTGAGGGACAGC-3′
tnf-α	5′-ATGAGAAGTTCCCAAATGGC-3′	5′-CTCCACTTGGTGGTTTGCTA-3′
pai-1	5′-GACACCCTCAGCATGTTCATC-3′	5′-AGGGTTGCACTAAACATGTCAG-3′
mmp3	5′-ACATGGAGACTTTGTCCCTTTTG -3′	5′-TTGGCTGAGTGGTAGAGTCCC-3′
sod1	5′-AACCAGTTGTGTTGTCAGGAC-3′	5′-CCACCATGTTTCTTAGAGTGAGG-3′
sod2	5′-CAGACCTGCCTACGACTATGG-3′	5′-CTCGGTGGCGTTGAGATTGTT-3′
sod3	5′-CCTTCTTGTTCTACGGCTTGC-3′	5′-TCGCCTATCTTCTCAACCAGG-3′
nox1	5′-AGTGATGTATGCAGCATTTACCA-3′	5′-CCATAGCTGACGTTACCATGAGA-3′
nox2	5′-CGCATGCCTTTGAGTGGTTT-3′	5′-ACGCCTATTGTGGTGTTAGGG-3′
nox4	5′-GAAGGGGTTAAACACCTCTGC-3′	5′-ATGCTCTGCTTAAACACAATCCT-3′
xo	5′-GAATGGCAAAAAGGTGGTGGA-3′	5′-AGCAACATGATGCAAGGAGC-3′
cd3e	5′-GACTATGAGCCCATCCGCAAA-3′	5’TAGGACACFGTGTTCACCAGGA-3’
foxp3	5′-GGCCCTTCTCCAGGACAGA-3’	5’GCTGATCATGGCTGGGTTGT-3’
